# The Ethics of the New Criminal Law Amendment Bill

**Published:** 1917-02-24

**Authors:** 


### THE PREVENTION OF VENEREAL DISEASE.

### The Ethics of the New Criminal Law Amendment Bill.

General approval has met Sir Bryan Donkin's appeal for the prevention of venereal disease. There is an impression, vague, nebulous, but widely diffused, an impression derived through Methodism from mediæval ecclesiasticism, that these diseases were "sent" as punishments for illicit sexual relations, and consequently that there would be a certain impiety in preventing, or trying to prevent them. Another objection to their prevention, an objection that is widely felt, though it is not often expressed, is that since the fear of these diseases acts as a deterrent to the illicit converse of the sexes, they ought not to be prevented lest this deterrent should be removed, and immorality should increase in consequence. There is not much reason in these objections, but it is undeniable that they are very widely entertained, and form such a barrier of prejudice that the Royal Commission on Venereal Diseases, though it dealt very thoroughly with the subject of treating them, never ventured to touch upon the matter of prevention. More than this, Sir Bryan Donkin has, we believe, encountered from the members of the Royal Commission and other influential persons a good deal of passive opposition to his proposal. It is worth while, therefore, to examine the grounds of the objections to prevention.

In the first place, if these diseases were indeed "sent" as a punishment for illicit sexual intercourse, there can be no objection to preventing their dissemination by legal marital relations. At present the rain falls alike upon the just and on the unjust, and the innocent spouse of a diseased person is just as likely to be infected as the guilty partner in an illicit connection. There can scarcely be any interference with the ways of Providence in saving from punishment those who have incurred no penalty. Rather it is a pious duty to safeguard the innocent; and though it is true that in practice we should not be able to safeguard the innocent without at the same time affording the guilty a means of immunity, and so presumably interfering with the designs of a Higher Power, yet it seems doubtful whether this interference would be different in principle from that which we already exert. For if venereal diseases are indeed a punishment for illicit sexual relations, and if it is impious to interfere with this punishment, the guilt of impiety is already incurred. It is already incurred by treating these diseases, by cutting them short, and ameliorating the sufferings that result from them. We are already engaged in searching for a remedy that shall be effectual in every case, and if such a remedy is found, it will surely be almost as effectual and as impious an interference with the punishment as if we had prevented the diseases.

The next objection is what may be termed the police objection. The fear of these diseases is, it is said, such an effectual deterrent against sin that if the fear were removed sexual immorality would straightway break out into wild excesses, and, if it did not actually become universal, would at any rate become very much more widespread. This is what is asserted, or, if it is not asserted, this is at the back of the minds of those very well-meaning people, members of the Royal Commission and others, who, if they do not actually oppose schemes of prevention, will take no part in promoting them and offer to them a passive resistance. The answer to this objection is twofold. In the first place, the alleged or supposed result of preventing venereal diseases is purely supposititious. It rests on no evidence, and is at the utmost a probability, and a probability whose degree we have no means of estimating. It is certain that at the present time there is a good deal of immorality in spite of the deterring effect that the diseases are supposed to exert. It is certain that there are great multitudes of both sexes who are continent on principle, and on whom the total abolition of the diseases, if it were possible, would not have the slightest effect, for it is not the risk of incurring the disease that keeps them moral. Between the two classes there may be a margin of persons who are kept straight by the fear of infection; but in the first place, we do not know for certain that there is any such margin; and in the second, if there be such a margin, we do not know how large or how small it is. It is scarcely reasonable to forgo a certain and immense benefit lest we should incur a very uncertain, and possible a very small, detriment.

Whether these diseases have been inflicted on mankind as a deterrent against contemplated immorality and as a punishment for actual immorality we do not know, and until we receive another revelation we never shall know. But it is legitimate to ask why, if it is so, the infliction of one at least of these diseases should have been delayed so long. We have ample evidence that sexual immorality has been prevalent in Europe for ages. It can scarcely be contended that, bad as the morals of our ancestors were in the fifteenth century, they were worse than those in the decadence of Rome, or in the later Greek Empire. Yet syphilis does not appear to have existed in Europe until it was introduced from America at the end of the fifteenth century by the companions of Columbus. This, however, is clear: that if it is impious, immoral, or *contra bonos mores* to prevent venereal disease, it must be equally impious, immoral, and *contra bonos mores* to cure it or to alleviate its symptoms. In so far as we diminish its effect as a punishment, in so far we diminish its effect as a deterrent.

There is one more aspect of the matter that can scarcely be passed over. The efforts of human legislators in the treatment of criminals have been for many years directed as much to their reformation as to their punishment. The design of the legislator is not so much to inflict pain as to effect reform. But supposing punishment to have been the purpose of the Divine Legislator in inflicting these diseases upon humanity, it is clear that reform had no place in the scheme. Once a man is infected with syphilis he is immune from re-infection, and therefore whatever deterrent effect the fear of infection may possess for the possible offender who is as yet free from disease, it can possess none for the infected. From him the motive for reform is effectually removed, and the dog is left to return to its vomit, the sow to her wallowing in the mire. It is not thus that human legislators frame their schemes for treating offenders against human laws, and we may well put to those that look upon venereal diseases in the light of punishments the question of Eliphaz the Temanite: "Shall mortal man be more just than God? Shall a man be more pure than his Maker?"

